# Oral-Gut-Brain Axis in Experimental Models of Periodontitis: Associating Gut Dysbiosis With Neurodegenerative Diseases

**DOI:** 10.3389/fragi.2021.781582

**Published:** 2021-12-10

**Authors:** Luis Daniel Sansores-España, Samanta Melgar-Rodríguez, Katherine Olivares-Sagredo, Emilio A. Cafferata, Víctor Manuel Martínez-Aguilar, Rolando Vernal, Andrea Cristina Paula-Lima, Jaime Díaz-Zúñiga

**Affiliations:** ^1^ Periodontal Biology Laboratory, Faculty of Dentistry, University of Chile, Santiago, Chile; ^2^ Faculty of Dentistry, Autonomous University of Yucatán, Mérida, México; ^3^ Department of Periodontology, School of Dentistry, Universidad Científica Del Sur, Lima, Perú; ^4^ Biomedical Neuroscience Institute, Faculty of Medicine, Universidad de Chile, Santiago, Chile; ^5^ Department of Neuroscience, Faculty of Medicine, Universidad de Chile, Santiago, Chile; ^6^ Institute for Research in Dental Sciences, Faculty of Dentistry, Universidad de Chile, Santiago, Chile; ^7^ Department of Medicine, Faculty of Medicine, University of Atacama, Copiapó, Chile

**Keywords:** periodontitis, Alzheimer’s disease, gut microbiota, dysbiosis, pathogen, keystone

## Abstract

Periodontitis is considered a non-communicable chronic disease caused by a dysbiotic microbiota, which generates a low-grade systemic inflammation that chronically damages the organism. Several studies have associated periodontitis with other chronic non-communicable diseases, such as cardiovascular or neurodegenerative diseases. Besides, the oral bacteria considered a keystone pathogen, *Porphyromonas gingivalis*, has been detected in the hippocampus and brain cortex. Likewise, gut microbiota dysbiosis triggers a low-grade systemic inflammation, which also favors the risk for both cardiovascular and neurodegenerative diseases. Recently, the existence of an axis of Oral-Gut communication has been proposed, whose possible involvement in the development of neurodegenerative diseases has not been uncovered yet. The present review aims to compile evidence that the dysbiosis of the oral microbiota triggers changes in the gut microbiota, which creates a higher predisposition for the development of neuroinflammatory or neurodegenerative diseases.The Oral-Gut-Brain axis could be defined based on anatomical communications, where the mouth and the intestine are in constant communication. The oral-brain axis is mainly established from the trigeminal nerve and the gut-brain axis from the vagus nerve. The oral-gut communication is defined from an anatomical relation and the constant swallowing of oral bacteria. The gut-brain communication is more complex and due to bacteria-cells, immune and nervous system interactions. Thus, the gut-brain and oral-brain axis are in a bi-directional relationship. Through the qualitative analysis of the selected papers, we conclude that experimental periodontitis could produce both neurodegenerative pathologies and intestinal dysbiosis, and that periodontitis is likely to induce both conditions simultaneously. The severity of the neurodegenerative disease could depend, at least in part, on the effects of periodontitis in the gut microbiota, which could strengthen the immune response and create an injurious inflammatory and dysbiotic cycle. Thus, dementias would have their onset in dysbiotic phenomena that affect the oral cavity or the intestine. The selected studies allow us to speculate that oral-gut-brain communication exists, and bacteria probably get to the brain via trigeminal and vagus nerves.

## Introduction

The human microbiome comprises a wide variety of genes composing the bacteria, viruses, funghi, and archea (Lloyd-Price et al., 2016; Dominguez-Bello et al., 2019). The microbiome plays a vital role in our development, physiology, immunity and nutrition (Dethlefsen et al., 2007). It is estimated that only the host’s cells and bacteria ratio is closer to 1:1, and not 1:10 as was described previously (Dethlefsen et al., 2007; Sender et al., 2016). Otherwise, the term microbiota is the collection of microbes such as bacteria, viruses, funghi, and archea, that cover all the body surfaces. Recently, it has been proposed that many chronic non-communicable diseases originate from an unbalanced microbiota (Dethlefsen et al., 2007; Hajishengallis et al., 2012; Lamont et al., 2018). Thus, the composition of the microbiota could predispose or favor the onset or progression of certain diseases or health conditions, such as insulin resistance, diabetes mellitus, dyslipidemia, periodontitis, and Alzheimer’s Disease (AD) (Turnbaugh et al., 2006; Cani et al., 2007; Aemaimanan et al., 2013; Komazaki et al., 2017; Jin et al., 2018; Díaz-Zúñiga et al., 2020). Thus, the imbalance of the microbiota known as dysbiosis, is defined as a compositional and functional alteration in the behavior of both one or a group of microorganisms due to quantitative or qualitative changes (Hajishengallis et al., 2012; Levy et al., 2017). Dysbiosis can be characterized by a loss or increase of certain microorganism, or by a loss of overall microbial diversity (Petersen and Round, 2014).

Several studies have shown that periodontitis affects cognitive status, and this effect was proposed to be a consequence of a chronic low-grade inflammatory state (Poole et al., 2015; Ishida et al., 2017; Wu et al., 2017; Ilievski et al., 2018; Zhang et al., 2018; Dominy et al., 2019; Díaz-Zúñiga et al., 2020; Kantarci et al., 2020). Periodontitis is an infectious bone-resorptive chronic disease caused by the dysbiosis of the periodontal microbiota (Hajishengallis et al., 2012; Hajishengallis, 2015). Among the bacteria associated with dysbiosis, the Gram-negative bacteria *Porphyromonas gingivalis*, *Aggregatibacter actinomycetemcomitans*, *Tannerella forsythia*, and *Prevotella intermedia* have been associated with its onset and progression (Hajishengallis et al., 2012; Hajishengallis and Lamont, 2012; Hajishengallis, 2014; Hajishengallis, 2015). These bacteria produce several virulence factors that give them the ability to induce host tissues’ invasion, adhesion, phagocytosis evasion, migration, and a consequent pro-inflammatory response in different tissues or organs (Perry et al., 1996; Laine and Winkelhoff, 1998; Foschi et al., 2006; Kim et al., 2007; Vernal et al., 2008; Darveau et al., 2012; Shimotahira et al., 2013; Vernal et al., 2014a; Vernal et al., 2014b; Díaz-Zúñiga et al., 2015a; Díaz-Zúñiga et al., 2015b; Doke et al., 2017). Periodontal dysbiotic bacteria have also been detected in other distal tissues, such as *decidua basalis* of the placenta, intima layer of atherosclerotic plaques, in the hippocampus of people who died due to AD, and in the feces of people with ulcerative colitis (Riviere et al., 2002; Poole et al., 2013; Szulc et al., 2015; Vanterpool et al., 2016; Udagawa et al., 2018; Dominy et al., 2019; Fischer et al., 2019). Although it has recently been recognized that the oral cavity and the intestine are connected by the microbiota and that a bidirectional relationship may exist, it is still unclear whether oral dysbiosis can affect both gut microbiota and the brain. In this way, the present review aims to determine if the dysbiosis of the oral microbiota trigger changes in the gut microbiota, creating a higher predisposition for the development of neuroinflammation or neurodegenerative diseases.

## Oral and Gut Communication

Patients affected by periodontitis can have an area of ​​ulceration of 20 cm^2^ within their periodontium (Hujoel et al., 2001). Interestingly, periodontitis has been linked to other pathological conditions by causing transient bacteremia (Schenkein et al., 2000; Komine-Aizawa et al., 2019). Although it is known that the presence of bacteria in the bloodstream causes sepsis, there is no evidence of sepsis due to periodontal bacteria in the bloodstream. Leukocytes cannot recognize, fix, and engulf bacteria in high-velocity liquids such as the bloodstream (Minasyan, 2014). However, bacteria can be attracted (by electrical charges) to the erythrocytes’ surface and can be killed by contact through the release of oxygen from the oxyhemoglobin (Minasyan, 2017a). If bacteria survive this oxidative attack, they are consecutively filtered in the liver and spleen (Minasyan, 2017a). In some cases, bacteria can overload the liver and the spleen, and induce hepato- or splenomegaly (Minasyan, 2017a). Also, it has been proposed that bacteria enter the erythrocytes by creating membrane pores, and once inside these cells, they can be killed by oxidation or either be resistant (Minasyan, 2014; Minasyan, 2016). Only bacteria capable of resisting this outbreak would cause host death by sepsis (Minasyan, 2016). In this sense, once in the bloodstream, oral bacteria are rapidly killed by erythrocytes or filtered at the liver level so that transient bacteremia could induce a low-grade inflammatory liver response, and thus influence other tissues or organs by cytokine secretion (Minasyan, 2017b).

**FIGURE 1:**
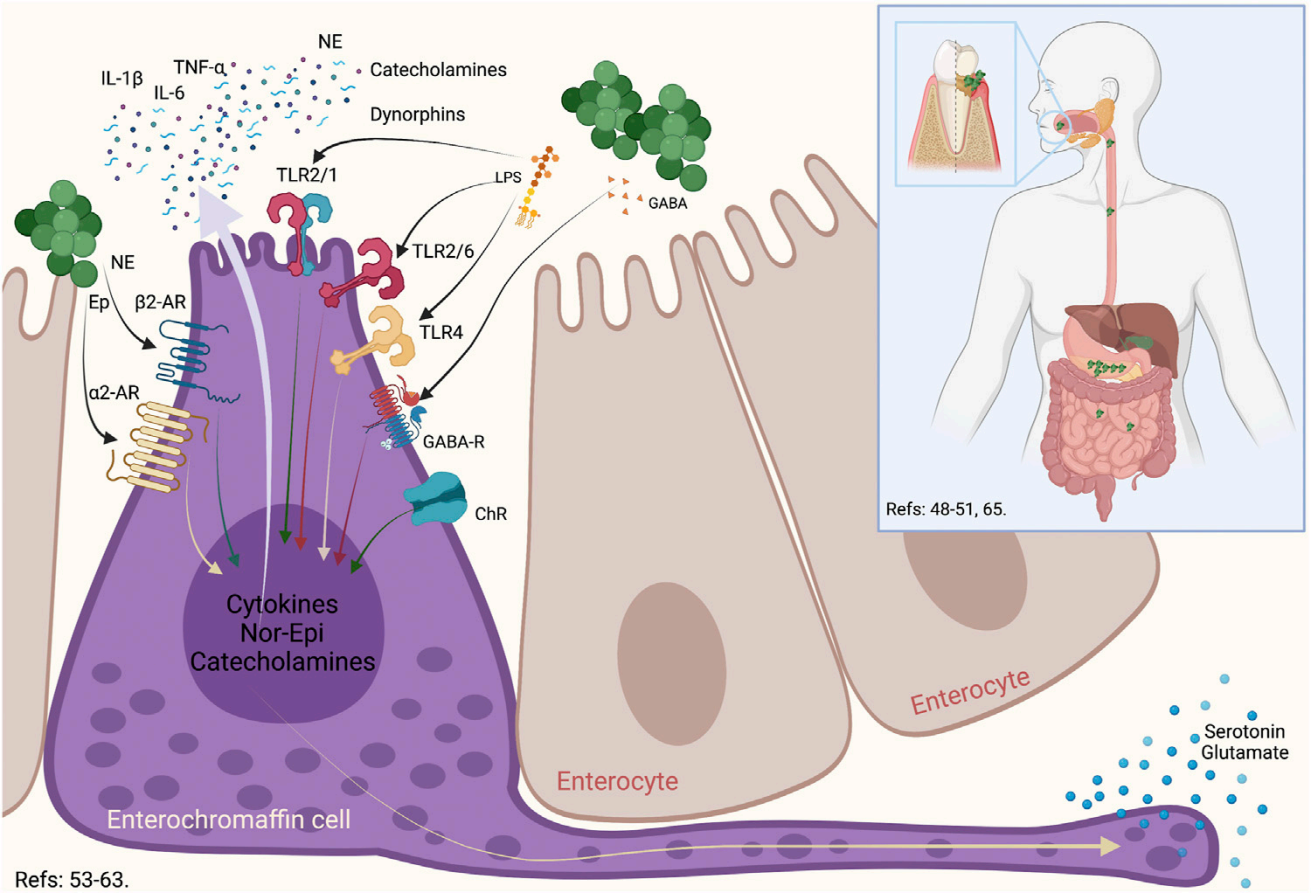
Enterochromaffin cell role in the oral-gut communication. During an oral dysbiosis, the increase in anaerobic bacteria associated with periodontitis produces an increase in the swallowed bacterial load. These bacteria, upon reaching the intestine, can generate an imbalance in the gut microbiota. One of the cells that is capable of recognizing both its own bacteria and bacteria that are relevant to the gut microbiota are the enterochromaffin. These cells possess a series of surface receptors, such as TLR2, TLR4, GABA, ChR, α2-AR, and β2-AR, which allows this cell to respond to a wide variety of bacteria, their virulence factors or even, the neurotransmitters that some of them produce. In this way, normobiosis or dysbiosis differentially activates enterochromaffin cells, which through glutamate or serotonin stimulate efferent vagal neurons to have an associated response. TLR: Toll Like Receptor, GABA: γ-Amino Butyric Acid, ChR: Cholinergic receptor, α2-Adrenergic Receptor, β2-Adrenergic Receptor, NE: Nor-Epinephrin, Ep: Epinephrin, IL: Interleukin, TNF: Tumoral Necrosis Factor.

It is noteworthy to mention that recent studies have shown that the amount of bacteria present in the saliva is about 106/ml, which means that a person regularly swallows around 1012–1013 bacteria *per* day (von Troil-Lindén et al., 1995; Boutaga et al., 2007; Saygun et al., 2011; Arimatsu et al., 2014). After inoculating 10^9^ CFU of *P. gingivalis* by oral gavage, at 3 h, this bacterium was detected in the ileum and at 16 h in the colon, inducing gut dysbiosis shortly after being ingested (Arimatsu et al., 2014). Thus, the presence of periodontitis and the consequent swallowing of high loads of anaerobic bacteria could generate imbalances in the gut microbiota (Arimatsu et al., 2014). In this context, a particular cell type in the intestine, the enterochromaffin cells, permanently senses the invading pathogenic bacteria (Bellono et al., 2017). The dysbiosis-associated bacteria can be recognized by the enterochromaffin cells, which release cytokines and neurotransmitters to the afferent fibers of the vagus nerve and induce an intestinal sympathetic response (Figure 1) (Raybould et al., 2004).

Generally, the enterochromaffin cells are the most abundant endocrine cells in the intestine and are distributed widely from the stomach to the rectum (Gunawardene et al., 2011). The primary role of enterochromaffin cells is to synthesize, store, and secrete serotonin (Bellono et al., 2017). Also, enterochromaffin cells produce the corticotropin-releasing hormone, cholecystokinin, and somatostatin in response to virulence factors of pathogenic bacteria (Gershon and Tack, 2007; Hansen and Witte, 2008). In general terms, enterochromaffin cells have an essential role as a regulator of the secretion and motility of the intestine, and are considered a chemosensory to modulate neural pathways (Bellono et al., 2017). It has been demonstrated that these cells express on their surface the pituitary adenylate-cyclase-activating peptide, α-adrenergic, β-adrenergic, cholinergic, corticotropin-releasing hormone, and γ-aminobutyric acid (GABA) receptors, and can respond to neurotransmitters secreted by the gut microbiota (Gershon and Tack, 2007; Hansen and Witte, 2008). These cells also produce catecholamines, dynorphins, norepinephrine, and cytokines to the intestinal lumen in order to restore homeostasis, favoring the growth of commensal bacteria and inducing the death of pathogenic bacteria (Alonso et al., 2008). Any variability in the quality or quantity of the gut microbiota can be rapidly sensed by the enterochromaffin cells through Toll-like receptors (TLR) that recognize certain virulence factors (Wheatcroft et al., 2005; Bogunovic et al., 2007; Hansen and Witte, 2008). After stimulation, enterochromaffin cells secrete serotonin that the afferent nerves recognize to establish synapsis with these cells (Rhee et al., 2009). The vagus nerve fibers possess serotonin receptors and are located in the proximity of these cells (Raybould et al., 2004). In response, the vagus nerve increases intestinal motility and permeability, stimulates mucous secretion, induces diarrhea, and triggers intestinal inflammation (Mayer, 2000). Consequently, the intestinal sympathetic response allows macrophages and mast cells to migrate due to the increased intestinal permeability and bacteria invasion (Rhee et al., 2009). Finally, considering their dual role as endocrine and excitatory, the enterochromaffin cells have also been named neuropodal cells (Kaelberer et al., 2018).

## Experimental Periodontitis Induces Gut Dysbiosis, Intestinal Barrier Permeabilization, and Inflammation

**FIGURE 2:**
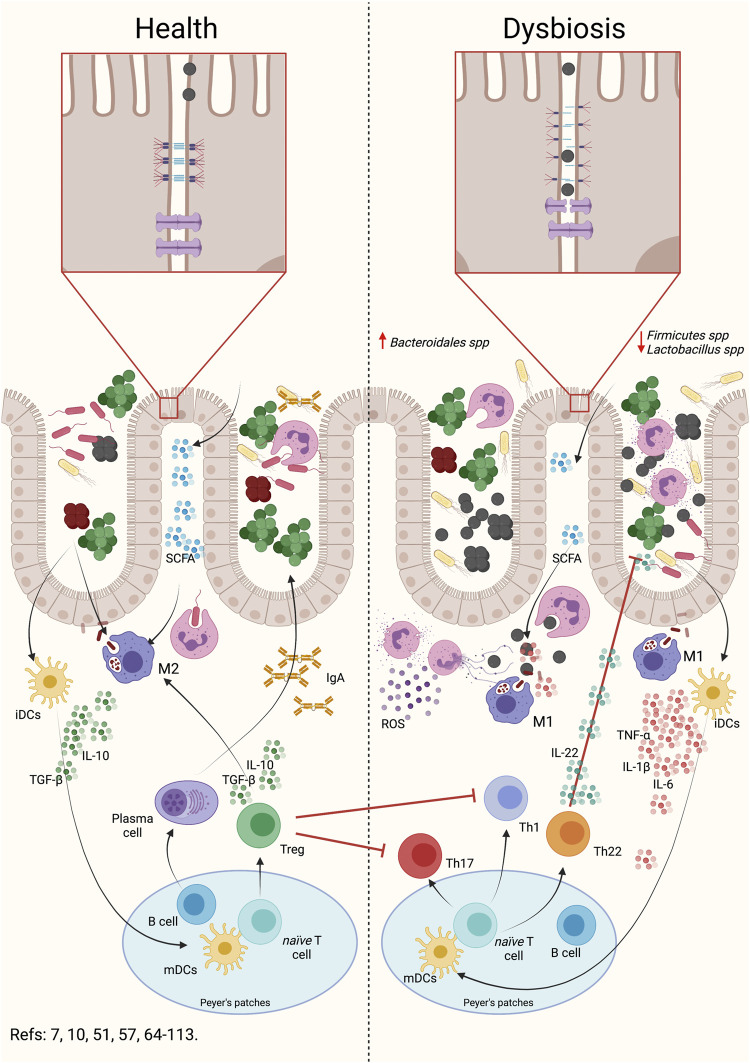
Oral dysbiosis induce gut dysbiosis, intestinal barrier permeabilization and intestinal inflammation. During intestinal dysbiosis caused by periodontal anaerobic bacteria, there is an alteration in the Bacteroidales spp and Firmicutes spp rate. Intestinal dysbiosis produces an alteration in SCFA, increasing acetate and propionate and decreasing butyrate. This alteration can induce an activation of the inflammatory response in macrophages and neutrophils. As a consequence, dysbiosis triggers the permeabilization of the intestinal barrier by the rearrangement of the adherent junctions. This reorganization implies a modification in the location and number of these junctions, which allows bacteria or their virulence factors to enter the submucosa. Once in the submucosa, the primary cells of the immune system will engulf the bacteria and respond by secreting pro-inflammatory cytokines. Depending on the bacterial load or virulence factors that are entering, the primary immune cells may secrete chemokines, which attract dendritic cells. Dendritic cells will engulf the antigen, process it, internalize it, and present to CD4^+^ T lymphocytes in Peyer’s patches or regional lymph nodes. Subsequently and, depending on the antigen presented, the clonal expansion and differentiation of the CD4^+^ T lymphocytes to the different effector phenotypes will occur. The presence of Th1 and Th17 lymphocytes will be associated with a higher pro-inflammatory response and permeabilization of the intestinal barrier. Evidence suggests that the presence of Th22 lymphocytes and IL-22 would influence the proliferation of anaerobic bacteria, participating in the modulation of the permeabilization of the barrier. In addition, the presence of Treg lymphocytes will decrease the inflammatory response, allowing the recovery of intestinal homeostasis. Indeed, during normobiosis, bacteria or their factors can be internalized into the submucosa by the epithelial cells themselves and, when recognized by the primary immune cells, differentiate into modulating phenotypes. This modulating response is characterized by the secretion of modulating or regulatory cytokines such as IL-10 or TGF-β1. Dendritic cells will be able to recognize antigens and present them to T or B lymphocytes, which will proliferate and differentiate into Treg lymphocytes or plasma cells, which will modulate the intestinal response, maintaining homeostasis. SCFA: Short chain fat acids, Th: T helper lymphocytes, Treg: T regulatory lymphocytes, IL: Interleukin, TGF-β1: Transforming growth factor β1, TNF: tumor necrosis factor, iDCs: immature dendritic cells, mDCs: mature dendritic cells, IgA: Immunoglobulin A, ROS: reactive oxygen species.

Several studies using experimental periodontitis induced by *P. gingivalis* oral gavage demonstrated intestinal inflammatory events characterized by changes in the gut microbiota composition, intestinal barrier permeability, and the modulation of the intestinal immune response (Figure 2) (Arimatsu et al., 2014; Nakajima et al., 2015; Sato et al., 2017; Sato et al., 2018).

### Gut Dysbiosis

The pathogenic colonization of the intestine by anaerobic oral bacteria induce an imbalance in the normal gut microbiota (Figure 2) (Arimatsu et al., 2014; Nakajima et al., 2015; Komazaki et al., 2017; Jia et al., 2018; Lourenço et al., 2018; Sato et al., 2018; Ohtsu et al., 2019; Feng et al., 2020; Hamamoto et al., 2020; Kobayashi et al., 2020). One of the main effects of *P. gingivalis* or *A. actinomycetemcomitans* oral gavage is to cause an imbalance in the *Firmicutes*/*Bacteroidetes* ratio in the gut microbiota (Arimatsu et al., 2014; Nakajima et al., 2015; Komazaki et al., 2017; Sato et al., 2017; Jia et al., 2018; Kato et al., 2018; Sato et al., 2018; Ohtsu et al., 2019; Hamamoto et al., 2020; Huang et al., 2020; Kobayashi et al., 2020). *Firmicutes* and *Bacteroidetes* species are the main components of the human bacterial microbiota, as revealed by gene sequencing of human fecal samples (Mariat et al., 2009). The *Firmicutes* are a *phylum* composed mostly of Gram-negative bacteria in which *Clostridium* and *Lactobacillus* are the most prevalent of the gut microbiota (Cassir et al., 2016). *Clostridium butyricum* can maintain the balance of the gut microbiota by its production of butyrate, which inhibits the growth of pathogenic *Escherichia coli*, preventing its binding to other intestinal bacteria (Wang et al., 2018a; Hagihara et al., 2020). Also, the butyrate level in the intestine is necessary to maintain a homeostatic T regulatory (Treg) response (Kurita-Ochiai et al., 1995; Atarashi et al., 2013).

On the other hand, *Bacteroidetes* are Gram-negative bacteria, from which *Bacteroides* and *Porphyromonas* are the most prevalent in the gut microbiota (Ley et al., 2005). Together with the *Firmicutes,* they represent 90% of the bacteria that make up the gut microbiota (Qin et al., 2010). *Bacteroidetes* and *Firmicutes* generate short-chain fat acids, acetate, propionate, and butyrate, which are anaerobic fermentation products (van de Wouw et al., 2017). Remarkably, acetate, propionate and butyrate modulate the acetylation and methylation of histones, which regulate gene expression on the host’s intestinal cells (Wang et al., 2018b). Thus, the increase in the production of short-chain fatty acids such as acetate by gut bacteria could modify host gene expression, causing a dangerous increase in gene expression associated with a pro-inflammatory response (Rousseaux and Khochbin, 2015). Conversely, the increase in butyrate plays a protective role in maintaining the intestinal barrier’s permeability by preventing the secretion of Interleukin (IL)-17 and triggering the different Treg subsets differentiation (Kurita-Ochiai et al., 1995; Wang et al., 2015; Jia et al., 2018; Szentirmai et al., 2019).

Also, a decrease in *Clostridium* and *Lactobacillus* was detected when *P. gingivalis* colonizes the intestine (Nakajima et al., 2015; Kobayashi et al., 2020). In this way, the presence of *P. gingivalis* in the intestine by itself increases lactic and n-butyric acids (Alonso et al., 2008; Rhee et al., 2009). Butyric acid induces the secretion of α-defensin in enterochromaffin cells, which play a central role in secreting antimicrobial peptides (Kaelberer et al., 2018).

### Intestinal Permeability

Another way oral bacteria could induce intestinal permeability changes is by altering intestinal permeability (Figure 2). In the experimental periodontitis induced by oral gavage using *P. gingivalis*, an increase in the concentration of *P. gingivalis* in the feces was not detected, which may indicate that this bacterium persists in the gut microbiota or spread to the intestinal connective tissue (Arimatsu et al., 2014; Nakajima et al., 2015; Hamamoto et al., 2020). Besides, experimental periodontitis induces the permeabilization of the intestinal barrier, which generates an increase in endotoxins at the serum level (Nakajima et al., 2015; Sato et al., 2018; Xue et al., 2020). In the first instance, *P. gingivalis* in the intestine is related to a significant decrease in the expression of proteins of the adherent zonula of the enterocytes, such as tight junction protein 1 (tjp1), claudin-1, and occludin (Nakajima et al., 2015; Xue et al., 2020). The *tjp1* encodes the zonula-occludens 1 (ZO-1) protein, essential for establishing tight junctions (Mohandas et al., 1995). These tight junctions also polarize the enterocyte by establishing an apical zone where the claudin-occludin-ZO1 complex is located, differentiating it from the basal area attached to the basal lamina (Massey-Harroche, 2000). When zonulin—a regulator of intestinal permeability molecule, also named pre-haptoglobin 2— is secreted to the intestinal lumen by enterocytes in response to microbiota changes, it triggers the polymerization of actin and the dissembling on the tight junctions by the action of protein kinase C (Asmar et al., 2002; Fasano, 2011). Increased serum zonulin levels are accompanied by a leaky intestinal barrier, dysbiosis and inflammation (Tajik et al., 2020). Interestingly, some bacteria, such as *E. coli* and *Prevotella spp,* cause zonulin release once recognized by enterocytes (Sturgeon and Fasano, 2016; Ciccia et al., 2017). Zonulin is recognized by the epidermic growth factor receptor (EGFR) and also transactivates proteinase-activated receptor (PAR)-2 (Fasano, 2011). Under gut dysbiosis, zonulin levels increase in the intestinal lumen, inducing the modification and re-distribution of tight junctions, which allows the increase of paracellular permeability (Gottardi et al., 1996; Asmar et al., 2002; Sturgeon and Fasano, 2016). If gut dysbiosis is persistent, the virulence factors or bacteria that invaded the connective tissue trigger the host immune response, which will increase the presence of pro-inflammatory mediators, with the consequent increase in intestinal permeability (Cani et al., 2007).

### Inflammation

Another effect of the intestinal colonization of periodontal bacteria is a noticeable intestinal inflammatory response (Figure 2). When gut dysbiosis occurs in the intestine due to increased periodontal pathogens and the alteration of intercellular junctions, the bacteria or their virulence factors spread through the paracellular pathway towards the underlying connective tissue (Gottardi et al., 1996; Asmar et al., 2002; Sturgeon and Fasano, 2016). There, they are quickly recognized by neutrophils or macrophages (Fournier and Parkos, 2012). Both neutrophils and macrophages can recognize bacteria through various receptors, such as CD14, TLR2, and TLR4 (West et al., 2011; Park and Lee, 20113; Futosi et al., 2013). The activation of these receptors is associated intracellularly with the activation of the transcriptional factor nuclear factor kappa B (NF-κB), which triggers the secretion of pro-inflammatory cytokines and chemokines (Zheng et al., 2010; West et al., 2011; Müller et al., 2014; Moreira Lopes et al., 2020). If the bacterium is of high virulence, it can induce the activation of a pro-inflammatory phenotype of neutrophils. This pro-inflammatory phenotype is characterized by the secretion of IL-1β, IL-6, and tumor necrosis factor (TNF)-α, the increase of myeloperoxidase levels, the release of reactive oxygen species (ROS), an increased phagocytosis capacity, and the release of the neutrophils extracellular trap (NET) associated to NETosis cell death (Sperandio et al., 2003; Jang et al., 2018; McVey Neufeld et al., 2019; Moreira Lopes et al., 2020). NETosis will favor the retention of invading bacteria, facilitating their phagocytosis by neutrophils or macrophages (Hahn et al., 2016). The cytokines secreted by neutrophils and the individual virulence of each bacterium may induce macrophage differentiation towards a pro-inflammatory or M1 phenotype (Mills et al., 2000). Besides, in the presence of gut dysbiosis, an increase in infiltrating of T helper (Th)1, Th17, and Th22 lymphocytes subsets is evidenced (Spiller, 2008; Ivanov et al., 2009; Hill and Artis, 2010). Curiously, the lipopolysaccharide (LPS) of *Bacteroides fragilis* and *Clostridium spp* can stimulate the production of IL-10 and regulate the Treg response, favoring the re-composition of gut microbiota by the local immune-suppression (Atarashi et al., 2013; Ramakrishna et al., 2019). Nevertheless, in experimental periodontitis models, an increase in Th1 and Th17, and decreased in Tregs infiltrating lymphocytes were observed (Atarashi et al., 2017; Sato et al., 2017; Sato et al., 2018). These changes in the immune response can be due to both an increase in pathogenic bacteria or a decrease in commensal bacteria (Nakajima et al., 2015).

In general, when pathogenic oral bacteria increases in dysbiotic diseases such as periodontitis, its detection in the bloodstream may be due to direct spreading from periodontal connective ulcerated tissue or by the permeabilization of the intestinal barrier through the bloodstream (Cani et al., 2007; Poveda-Roda et al., 2008).

## Enteric Nervous System and the Gut Microbiota Link

**FIGURE 3:**
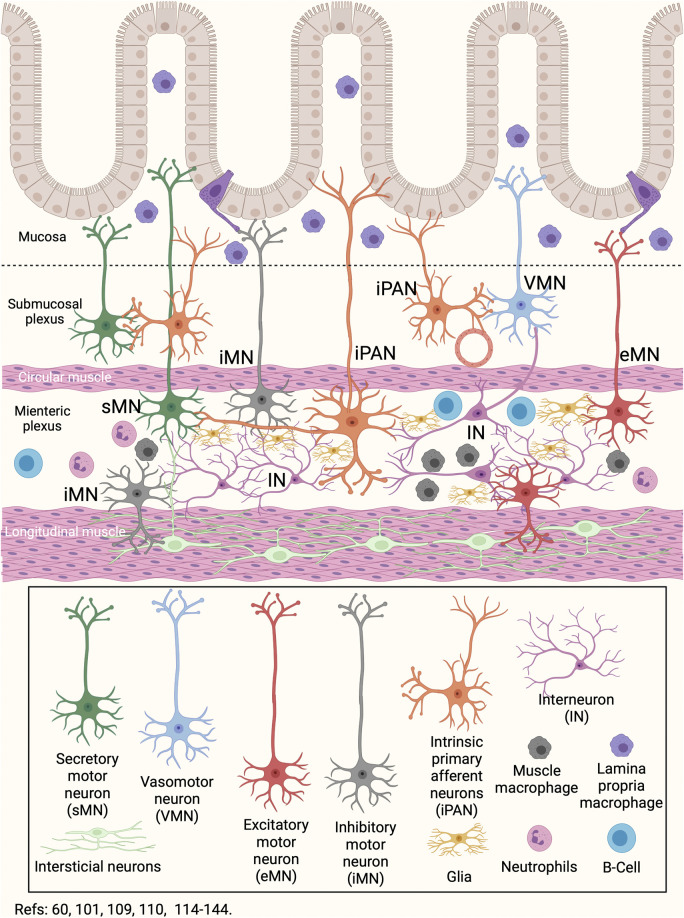
Gut-Brain neuroimmune communication. The network of neurons that innervates the intestine involves a series of neurons with different locations and functions. Secretory motor neurons, primary intrinsic afferent neurons, and vasomotor neurons are located in the submucosal plexus. In the myenteric plexus, the interneurons, the inhibitory, excitatory, secretory motor, and the intrinsic primary afferent neurons are located, in addition to the glia. In both the submucosal and myenteric plexus there are resident macrophages and neutrophils. In particular, myenteric plexus macrophages have the ability to migrate to the submucosa and regulate the neuronal and immune response induced by changes in the gut microbiota or food ingestion. Besides, the B cells can generate humoral responses at the myenteric plexus level. Finally, in the longitudinal musculature there are interstitial neurons. In this way, changes in the lumen are sensed by immune cells or neurons present in the mucosa and submucosa, and are regulated by neurons and immune cells located in the myenteric plexus.

There is a close link between the enteric nervous system and the gut microbiota (Veiga-Fernandes and Pachnis, 2017). Indeed, the development of the enteric nervous system is modulated by the gut microbiota (Veiga-Fernandes and Pachnis, 2017). TLR2 and TLR4 are expressed on enteric nervous cells and, when activated, increase the expression of glial-derived neurotrophic factor (GDNF), released against bacterial challenge (Anitha et al., 2012; Heiss and Olofsson, 2019). During growth, development and maturation, the enteric neurons and glial cells constantly sense intestinal bacteria (Heiss and Olofsson, 2019). Also, the smooth muscle cells express TLR2, TLR3, TLR4, and TLR9, and together with enteric neurons, are involved in the response against bacteria (Anitha et al., 2012; Brun et al., 2013; Kobayashi et al., 2019). Enteric neurons and intestinal macrophages have established critical crosstalk that communicates the immune and nervous systems (Figure 3) (Müller et al., 2014). Intestinal macrophages are distributed in the *lamina propria*, submucosal layer, and muscular plexus of the intestine (Gabanyi et al., 2016). The muscle macrophage function depends on both exogenous signals such as virulence factors of bacteria, viruses, or fungi, and endogenous molecules, such as damage-associated molecular patterns (DAMPs) or stress molecules (Müller et al., 2014; Gabanyi et al., 2016). In the healthy intestine, muscle macrophages secrete bone morphogenic protein 2 —a growth factor for enteric neurons—, and maintain enteric nervous system homeostasis by engulfing senescent enteric neurons (Müller et al., 2014). The static position of muscle macrophages, primarily alongside neuronal cell bodies and nerve fibers, provides an interface for optimal crosstalk (Gabanyi et al., 2016). In addition, muscle macrophages express β2-adrenergic receptors on its surface, which allows them to have a neuroprotective role, similar to that described by microglia in the central nervous system (Figures 1, 3) (Huuskonen et al., 2004; Matteoli et al., 2014; Erny et al., 2015; Gabanyi et al., 2016; Ifuku et al., 2016). It is known that the β2-adrenergic signal blocked the immune response, while the α-adrenergic signal can stimulate it (Guereschi et al., 2013). The counterpart is due to the catecholamine release by enterochromaffin cells or neurons of the intestine. Also, the mesenteric lymph nodes are innervated by sympathetic fibers, which release epinephrine or nor-epinephrine to the immune cells that express β2-adrenergic receptors (del Rey and Besedovsky, 2008). These data suggest that the high production of norepinephrine in intestinal tissues—by the neurons from the celiac and superior-mesenteric ganglia—is related to the constant activation of muscle macrophages towards a modulatory phenotype of inflammation (M2) produced by macrophages residing in the *lamina propria* (Gabanyi et al., 2016). Thus, the macrophages of the *lamina propria* respond to bacteria or virulence factors entering the connective tissue, while the muscle macrophages respond to neuronal signals (Gabanyi et al., 2016). Besides, neutrophils and CD8^+^ T lymphocytes express more β2-adrenergic receptors than CD4^+^ T cells do not, so inflammation would be lacking of a Tregs response, which require more intense signals (del Rey and Besedovsky, 2008). Additional to macrophages, the mast cells in the gut submucosal and myenteric plexuses can respond to neuron-derived factors such as substance P, immune signals such as immunoglobulin (Ig)-E, and regulate both neuronal and immune cell activity through various mediators, including histamine, serotonin, and TNF-α release (Suzuki et al., 1995; van Diest et al., 2012).

The human gastrointestinal tract is colonized by 100 trillion microorganisms (Furness et al., 1999; Hill and Artis, 2010) and contains an effective neuroimmune barrier that constantly monitors and responds to potentially dangerous gut microbiota changes (Furness et al., 1999; Hill and Artis, 2010; Branzk et al., 2014). The intestinal barrier is in a dynamic balance, integrating inputs from the epithelium, immune cells, neurons of the enteric nervous system, enteric glia, and microbiota (Figures 1, 2, 3) (Rhee et al., 2009; Cryan and Dinan, 2012; Furness et al., 2014; Müller et al., 2014; Hergott et al., 2016; Strandwitz, 2018; Yu et al., 2020). This barrier is not impermeable, and some commensal species such as *B. fragilis* induce an immune response in the intestinal epithelium and thus control the bacterial gut balance (Zafar and Saier, 2021). The colonization of pathogenic bacteria induces the differentiation and local infiltration of Th17 subsets, which produce IL-17 and protect against infection by enteric pathogens (Ivanov et al., 2009). Commensal bacteria associated with the epithelium also activate the production of IL-22 and IgA in CD4^+^ T and plasma cells, respectively (Figure 2) (Hill and Artis, 2010; Gommerman et al., 2014; Ramirez et al., 2020). In the connective tissue, the bacteria are also recognized by infiltrating dendritic cells that migrate to the mesenteric lymph node and trigger the differentiation toward Th17 subsets (Hill and Artis, 2010). Besides, the activation of TLR2 promotes the expression of S100β1, inducible nitric oxide synthase, and GDNF in the enteric glia, which can promote IL-22 release (Brun et al., 2013; Benarroch, 2019). Several metabolites of intestinal bacteria are relevant not only for intestinal protection but also for the microbiota interactions with the immune and nervous systems (Dass et al., 2007; Jenkins et al., 2016; Szentirmai et al., 2019). The short-chain fatty acids (acetate, propionate, and butyrate) derived from the anaerobic fermentation of indigestible carbohydrates play an important role in counteracting inflammation and maintaining intestinal homeostasis (Figure 2) (Dass et al., 2007; Smith et al., 2013). *Lactobacilli spp* metabolizes dietary tryptophan in humans and generates indole ligands of aryl hydrocarbon receptors, expressed in Th22 cells (Trifari et al., 2009; Rothhammer et al., 2016). Also, several microbiota members produce neurotransmitter molecules and neuropeptides, such as serotonin, dopamine, GABA, and brain-derived neurotrophic factor (Strandwitz, 2018). Thus, bacterial products can stimulate epithelial cells to release molecules of signaling that will regulate the function of the enteric nervous system, establishing a communication between the gut microbiota and the brain.

## Gut and Brain Communication

Gut bacteria can interact with the brain by communicating with the mesenteric and vagus nerve afferent fibers (Rhee et al., 2009). Interestingly, some bacteria produce and secrete different neurotransmitters, inducing a nerve impulse in the neurons underlying the intestinal epithelium (Strandwitz, 2018; Cui et al., 2020). Commensal bacteria interact with enterocytes through the α2-receptor and can either increase or decrease the ability to eliminate pathogenic bacteria in the intestinal lumen (Rhee et al., 2009). Gut bacteria interact directly with afferent nerves in the presence of intestinal permeability caused by inflammation or stress (Turnbaugh et al., 2006; Bravo et al., 2012; Foster and McVey Neufeld, 2013; Sarkar et al., 2016). Besides, the enterochromaffin cells in the intestine can translocate bacteria from the lumen to the afferent neurons (Rhee et al., 2009).

Interestingly, it was proposed that the recomposition of the microbiota, in particular, the *Bifidobacterium* and *Lactobacillus genera*, would allow the regulation of serotonin levels in the brain and the re-establishment of the intestinal homeostasis by parasympathetic stimulation (Mayer, 2000; Leonard, 2006; Turnbaugh et al., 2007; Rhee et al., 2009). Thus, the existence of direct and bidirectional communication between the gut and the brain is recognized (Clarke et al., 2013; Sarkar et al., 2016; Müller et al., 2020). Indeed, the microbiota would play a neurodevelopmental role (Clarke et al., 2013). Germ-free animals have an exaggerated stress response compared to usually colonized animals, and stress is reversed by reconstituting the microbiota (Sudo et al., 2004). The incorporation of *Bifidobacterium* or *Lactobacillus* can benefit stressful or depressive alterations in both health conditions or illness (Desbonnet et al., 2008). In fact, probiotics can increase the bioavailability of tryptophan, a precursor of serotonin (Desbonnet et al., 2008).

One of the theories that explain the communication between the gut and the brain is the existence of parallel outputs (Rhee et al., 2009). The sympathetic and parasympathetic nerve fibers, the hypothalamic-pituitary-adrenal axis, and the endogenous pathways that regulate pain are the key regulators of gastrointestinal function (Rhee et al., 2009). These three parallel outputs can alter the microbiota by modifying the environment or by microbiota-host signaling. First, the sympathetic and parasympathetic systems regulate intestinal motility, secretion of acid, HCO3-, mucus, and the immune response (Mayer, 2000). As there is a stimulation of the sympathetic response, it can increase intestinal motility, affecting the processing of nutrients by the gut microbiota. On the contrary, a parasympathetic response decreases motility, associated with bacterial overgrowth in the small intestine (van Felius et al., 2003).

Second, enterochromaffin cells secrete catecholamines, serotonin, norepinephrine, dynorphin, and cytokines into the intestinal lumen in response to changes in both the concentration of nutrients or the bacteria’s balance up the gut microbiota (Rhee et al., 2009). Although it is not yet well understood how bacteria interact with the nervous system, it has been proposed that the concentration of short-chain fatty acids is directly related to the presence of a symbiotic microbiota (Müller et al., 2020). Although bacteria constitute a crucial node in the gut-brain axis’s bidirectional relationship, it has also been called the microbiota-gut-brain axis (Sarkar et al., 2016). In general terms, gut dysbiosis produced by oral bacteria derived from oral diseases could be linked to neuroinflammatory events in the brain through this interaction.

The gut microbiota and immune system are involved in gut-brain communication (Sharon et al., 2016; Shi et al., 2017). The pathogens-associated molecular patterns (PAMPs), such as LPS, activate enteric glial cells and neural afferents fibers (Pochard et al., 2018). The vagal and dorsal root ganglion afferent fibers innervating the intestine express TLRs and cytokine receptors, communicating local intestinal signals to the central nervous system (Furness et al., 2014). Studies on germ-free intestines of mice treated with antibiotics indicate that the products of gut microbiota are potent regulators of immune response in the central nervous system (CNS) and are essential for intestinal barrier maintenance (Hernández-Chirlaque et al., 2016). In this sense, the presence of short-chain fatty acids in the bloodstream promotes microglial maturation and differentiation, and the uptake increase of tryptophan metabolites activates aryl hydrocarbon receptors which modulate the astrocyte activation during inflammation (Erny et al., 2015; Rothhammer et al., 2016). Also, the small chain fatty acid reduces the expression of pro-inflammatory factors, including C-C chemokine ligand (CCL)-2, IL-6, TNF-α, and nitric oxide synthase in both astrocytes and microglia (Huuskonen et al., 2004; Cho et al., 2019).

Peripheral signals from immune cells and microbial products can also be transmitted to the CNS via sensory vagal neurons of the nodose ganglion and by nociceptive and visceroceptive neurons of the dorsal root ganglion (Ordovas-Montanes et al., 2015; Yu et al., 2020). The gastrointestinal tract also contains intrinsic primary afferent neurons that initiate local reflexes, contribute input signals to the CNS, and participate in host-microbe interactions (Lai et al., 2017). In addition to the receptors of voltage-dependent excitatory cells, these afferent nervous fibers express TLRs and receptors for cytokines, which include IL-1β, IL-6, IL-17, TNF-α, and prostaglandins, as well as other receptors for molecules released by immune cells (Ramirez et al., 2020). One of the most important effects of TLR priming in nerve fibers is the decrease in the excitatory threshold (Ramirez et al., 2020). Thus, the nerve fibers of the intestine can respond to both bacterial virulence factors or cytokines released by immune cells after pathogenic bacteria priming, making the gut-brain axis a most complex communication (Ramirez et al., 2020). After crosstalk between sensory neurons and immune cells, neurons modulate tissue inflammation through the release of substance P, the peptide related to the calcitonin gene, the vasoactive intestinal peptide, and other interacting molecules (Lai et al., 2017). The substance P induces immune cells to secrete pro-inflammatory cytokines and contributes to tissue repair (O’Connor et al., 2004). The peptide related to the calcitonin gene is present in the terminals that innervate Peyer’s patches and regulates maturation, proliferation, migration, antigen presentation, and cytokine production by lymphocytes (Lai et al., 2017). The abdominal vagal afferent fibers express receptors for IL-1β and are activated by the systemic administration of IL-1β (Ek et al., 1998; Holzer et al., 2004). Finally, the vagal afferent fibers transmit peripheral signals to the solitary tract nucleus, which relays this information to the brainstem and anterior areas of the brain that contain neurons that regulate immune and inflammatory responses (Cutsforth-Gregory and Benarroch, 2017).

## Oral and Brain Communication

**FIGURE 4:**
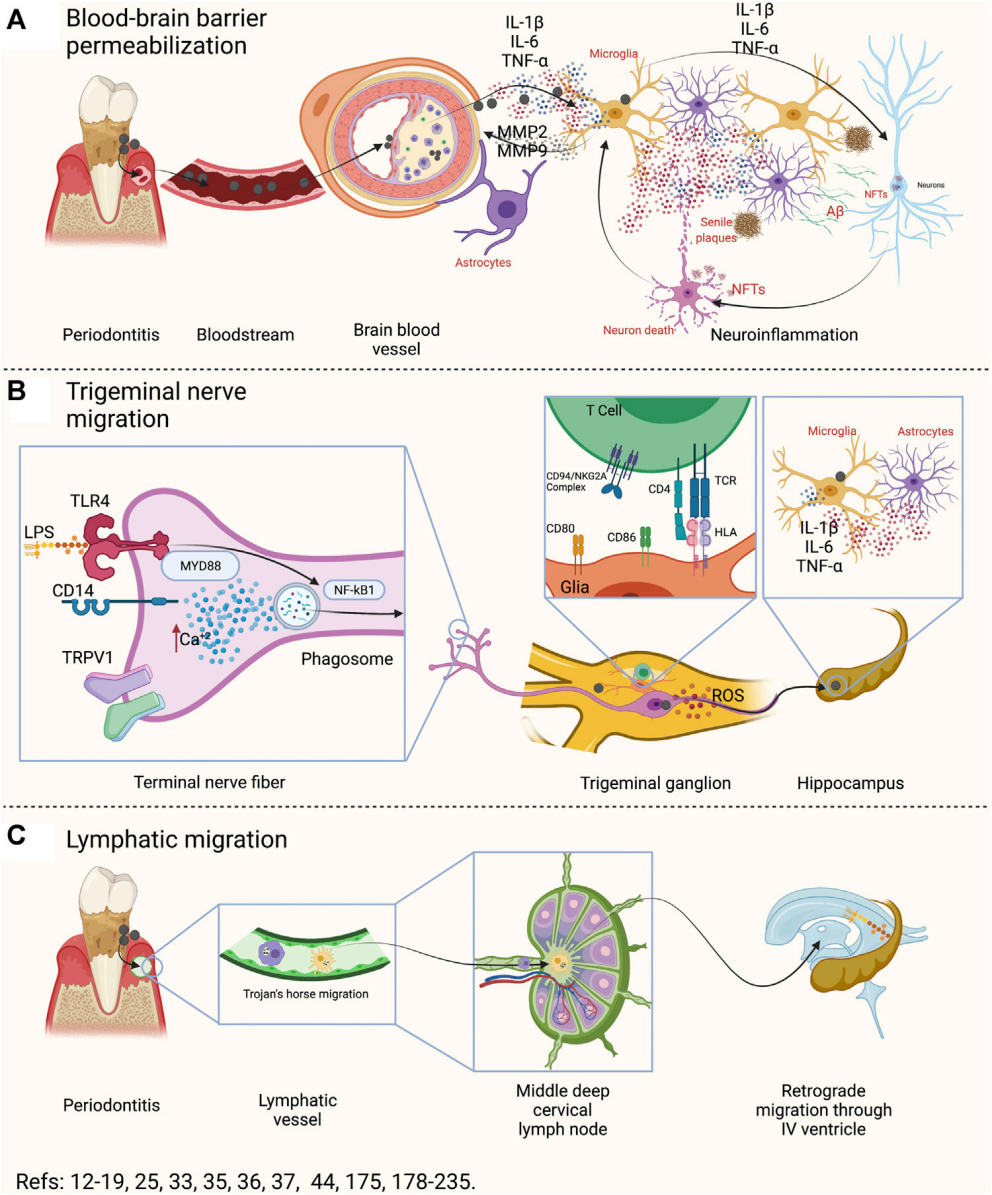
Oral-Brain axis components. **(A)** During periodontitis, possible transient bacteremia could induce atherogenesis at the brain level. Macrophages and endothelial cells associated with atheroma plaque can produce pro-inflammatory mediators, which spread to the brain. There, it produces functional changes both in the astrocyte of the neurovascular unit and in the microglia. Both cells, in response to local inflammation, will produce IL-1β, IL-6, IL-17, and TNF-α and, together with MMP2 and MMP9, will produce an exacerbation of inflammation and degradation of the proteins of the neurovascular unit. In this way, a breakout of the BBB will occurs. Then, the inflammation induced by microglia and reactive astrocytes will affect neuronal function, generating the necessary stimuli for the production of amyloid β and hyperphosphorylation of the Tau protein. In this way, the BBB breakdown may produce senile plaques, NFTs, and neuronal death. **(B)** The fibers of the trigeminal nerve that innervate the periodontal tissues have various surface receptors that can recognize LPS, capsular polysaccharides, fimbria, among other virulence factors. The activation of these receptors (TLRs, CDs, and TRPV1) can induce the activation of NF-κB, the formation of the phagosome or the increase of intracellular Ca+2. In response, the neuron will respond by producing IL-1β, IL-6, and TNF-α in the trigeminal ganglion. Furthermore, in the trigeminal ganglion there are glial cells that are capable of recognizing bacteria, engulfing, processing, and presenting them to the CD4^+^ T lymphocytes in the trigeminal ganglion. Pathogenic bacteria that have the ability to inhibit phage-lysosome formation can remain alive within the phagosome and, through vesicular trafficking, can move along the axon or dendrites of the neuron. Thus, this could be a possible bacterial migration pathway. Furthermore, neurons that recognize pathogenic bacteria could secrete pro-inflammatory cytokines in the trigeminal pontine nucleus or in other areas of the brain, and generate activation in microglia and astrocytes. **(C)** The lymphatic pathway is made up of antigen-presenting cells that recognize, incorporate, and process pathogenic bacteria, and migrate to the regional lymph node to present the antigen. In the regional lymph node they can present CD4^+^ T lymphocytes, which depending on the context, will differentiate into the different effector phenotypes. Certain pathogenic bacteria have the ability to inhibit phage-lysosome formation and thus survive and migrate utilizing host cell migration mechanisms. In this way, once in the lymph node, the phagocytes could migrate to another lymph node or, the bacteria could migrate through the lymphatic vessels to another lymphatic site. In particular, the III and IV cerebral ventricle drains, as do the submandibular or parotid lymph nodes, to the deep mid-cervical cervical. Therefore, the oral cavity and the brain would be lymphatically connected. IL: interleukin, TNF: tumor necrosis factor, MMP: matrix metalloproteinases, BBB: blood-brain barrier, LPS: lipopolysaccharide, TLRs: toll-like receptors, CDs: cluster of differentiation, TRPV1: transient receptor potential cation channel V1, NF-κB: nuclear factor κ B, NFTs: neurofibrillary tangles, TCR: T-cell receptor, HLA: human-leukocyte antigens, ROS: reactive oxygen species.

The oral cavity is one of the main entry routes for microorganisms, mainly bacteria (Li et al., 2000; Loesche and Lopatin, 1998). Some studies have detected *P. gingivalis* in the bloodstream after tooth brushing, flossing, or chewing food, in subjects with or without periodontitis, causing transient bacteremia (Li et al., 2000; Loos, 20015; Forner et al., 2006). This event allows oral bacteria to migrate and establish themselves in other tissues or organs, such as the intimal layer of the coronary arteries, the liver, or the placenta (Marcelino et al., 2010; Vanterpool et al., 2016; Komazaki et al., 2017; Udagawa et al., 2018; Fischer et al., 2019). Indeed, a recent study showed that the most prevalent bacterium colonizing the intima layer of both coronary and femoral arteries was *P. gingivalis* (Mougeot et al., 2017). Once in the bloodstream, bacteria induce an acute liver inflammatory phase response characterized by increased pro-inflammatory cytokines that could enter or, even without entering, influence brain function (Wu and Nakanishi, 2014; Wu et al., 2017; Ding et al., 2018; Ilievski et al., 2018; Zhang et al., 2018). Although the blood-brain barrier (BBB) generally prevents substances from entering the brain, molecules such as cytokines can enter through capillaries from the circumventricular organs by using specific cytokine transporters, increasing BBB’s permeability through transporters of brain endothelial cells (Pan and Kastin, 1999; Banks et al., 2002; Perry, 2004). In patients with AD, a BBB breakdown was associated with cognitive decline and inflammation (Bowman et al., 2018). Thus, the first potential link between oral and brain might be due to the breakdown of the BBB induced by the low-systemic inflammatory mediators in the bloodstream (Figure 4) (Hawkins and Davis, 2005).

### Blood-Brain Barrier Breakdown

Different studies in which the experimental periodontitis was induced through different methods, propose that periodontitis produces neuroinflammation and neurodegeneration (Poole et al., 2015; Ishida et al., 2017; Liu et al., 2017; Rokad et al., 2017; Singhrao et al., 2017; Wu et al., 2017; Ilievski et al., 2018; Zhang et al., 2018; Dominy et al., 2019; Díaz-Zúñiga et al., 2020; Kantarci et al., 2020). When analyzing if oral bacteria can enter the brain of people who are affected by AD, experimental and some descriptive studies have shown that it may be possible (Riviere et al., 2002; Foschi et al., 2006; Rokad et al., 2017; Singhrao et al., 2017; Han et al., 2019). To date, none of them has determined the possible pathway through which this bacterial entrance can occur.

Interestingly, transient bacteremia produced by *P. gingivalis* throughout a person’s life could lead to the formation of atheroma (Koren et al., 2011; Szulc et al., 2015; Bale et al., 2017). *P. gingivalis* secretes gingipains capable of cleaving the immune cell CD14 receptor, collagen type I and IV, fibrin, hemoglobin, laminin, among other extracellular matrix proteins (Sugawara et al., 2000; Tada et al., 2002; O'Brien-Simpson et al., 2009; Wilensky et al., 2013). Indeed, *P. gingivalis* can enter the intimal layer of the arteries and colonize them through the direct action of the fimbria attachment to the α5β1 complex, or by direct internalization of bacteria into endothelial cells (Imamura, 2003; Minasyan, 2014; Li et al., 2019). Once in the intima layer, the endothelial cells respond by activating NF-κB signaling and secreting pro-inflammatory mediators that allow the migration and chemotaxis of macrophages (Figure 4A) (Sorsa et al., 2004; Zhou and Windsor, 2006; Konradt and Hunter, 2018). Macrophages then internalize into the intima using changes in Ca^2+^ concentrations to destabilize endothelial cells’ tight junctions and act by direct cleavage of basal lamina by secreting matrix metalloproteinases (MMP)-2 and MMP-9 (Sorsa et al., 2004; Zhou and Windsor, 2006; Konradt and Hunter, 2018). This process creates a local inflammatory and oxidative phenomenon that will induce endothelial cells, fibroblasts, and macrophages to transform into foam cells (Chistiakov et al., 2017). Also, the mediators produced by macrophages, endothelial cells or DAMPs, will spread to the brain, being recognized by astrocytes (Figure 4A) (Norden et al., 2016; Bowman et al., 2018). Additionally, the pro-inflammatory cytokines will induce the activation of reactive-astrocytes, which will also produce MMP-2 and MMP-9, which will cleave agrin, β-dystroglycan, and laminins—that constitute the basal lamina to which astrocytes anchor to form the neurovascular unit—, inducing their uncoupling and with it, the breakdown of the BBB (Hawkins and Davis, 2005; Jaeger et al., 2009; Puntambekar et al., 2011; Rochfort et al., 2014; Song et al., 2015; Ueno et al., 2016; Rokad et al., 2017; Singhrao et al., 2017; Bowman et al., 2018). These phenomena trigger CCL2 to create a chemotactic gradient that will allow the entry of macrophages to engulf the metabolites derived from basal lamina cleavage (Puntambekar et al., 2011). Reactive astrocytes will recognize the cytokines released by their surface receptors and produce more IL-1β, IL-6, and TNF-α, polarizing the microglia toward a pro-inflammatory M1 phenotype (Figure 4A) (Block and Hong, 2005; Perry et al., 2010; Frost and Li, 2017). M1 microglia will secrete more MMP-2 and MMP-9 to permeabilize the BBB and favor the elimination of pro-inflammatory brain mediators (Song et al., 2015; Bowman et al., 2018). However, as there is a chronic origin of mediators, the breakdown creates a vicious cycle with the harmful effects of the permanent permeabilization of this BBB. In this context, there exist evidence showing that people affected by neurodegenerative diseases, such as AD have a dysfunctional BBB (Pan and Kastin, 1999; Kebir et al., 2007; Song et al., 2015; Carter, 2017). Nevertheless, neither in patients affected with periodontitis nor in experimental periodontitis models have been proven the existence of a dysfunctional BBB. Thus, there is no evidence showing if the BBB breakdown is before or after periodontitis, so further studies in this field are necessary to determine the role of periodontitis.

### Bacteria Migration Through Trigeminal Nerve Endings

Regarding the migration of oral bacteria through the nerve pathway to the brain, there is evidence of non-oral spirochetes such as *Borrelia burgdorferi* or *T. pallidum* identified in both the axons of peripheral nerves and within the brain of experimental animals (Sell and Salman, 1992; Cadavid et al., 2000; Miklossy, 2016). Although there is little published evidence that oral spirochetes can invade nerve tissues, *T. denticola* was detected in the trigeminal ganglion, the pontine nucleus of the trigeminal nerve, and hippocampus in both subjects who died due to AD and in mice affected by endodontic lesions (Rupf et al., 2000; Riviere et al., 2002; Foschi et al., 2006). These findings raise the possibility that *T. denticola* enters the brain using the peripheral endings of the trigeminal nerve. Even though the entrance origin cannot be determined from these studies, the results suggest that most *Treponema spp* can invade the central and peripheral nervous systems (Riviere et al., 2002). Recently, the LPS of both *E. coli* and *P. gingivalis* were shown to activate TLR4 and a type of transient receptor potential (TRP) in trigeminal nerve endings and supporting non-neuronal cells (Figure 4B) (Diogenes et al., 2011; Meseguer et al., 2014; Kaewpitak et al., 2020). The TRPs are channels located in the nociceptors, including TRPA1 and TRPV1, activated in response to bacterial infection (Chung et al., 2011; Gibbs et al., 2011; Huang et al., 2012). TRPV1 is expressed in 20–35% of trigeminal neurons, and TRPA1 is expressed in the trigeminal ganglion in approximately 6–10% of neurons (Gibbs et al., 2011; Huang et al., 2012). The LPS of *P. gingivalis* activates the trigeminal neurons through the TRPA1 and TLR4-dependent pathways (Diogenes et al., 2011; Meseguer et al., 2014; Kaewpitak et al., 2020). When TLR4 is activated in neurons, intracellular Ca^2+^ levels increase and activate TRPA1, creating a positive enhancement of Ca^2+^ signaling (Zurborg et al., 2007).

**FIGURE 5:**
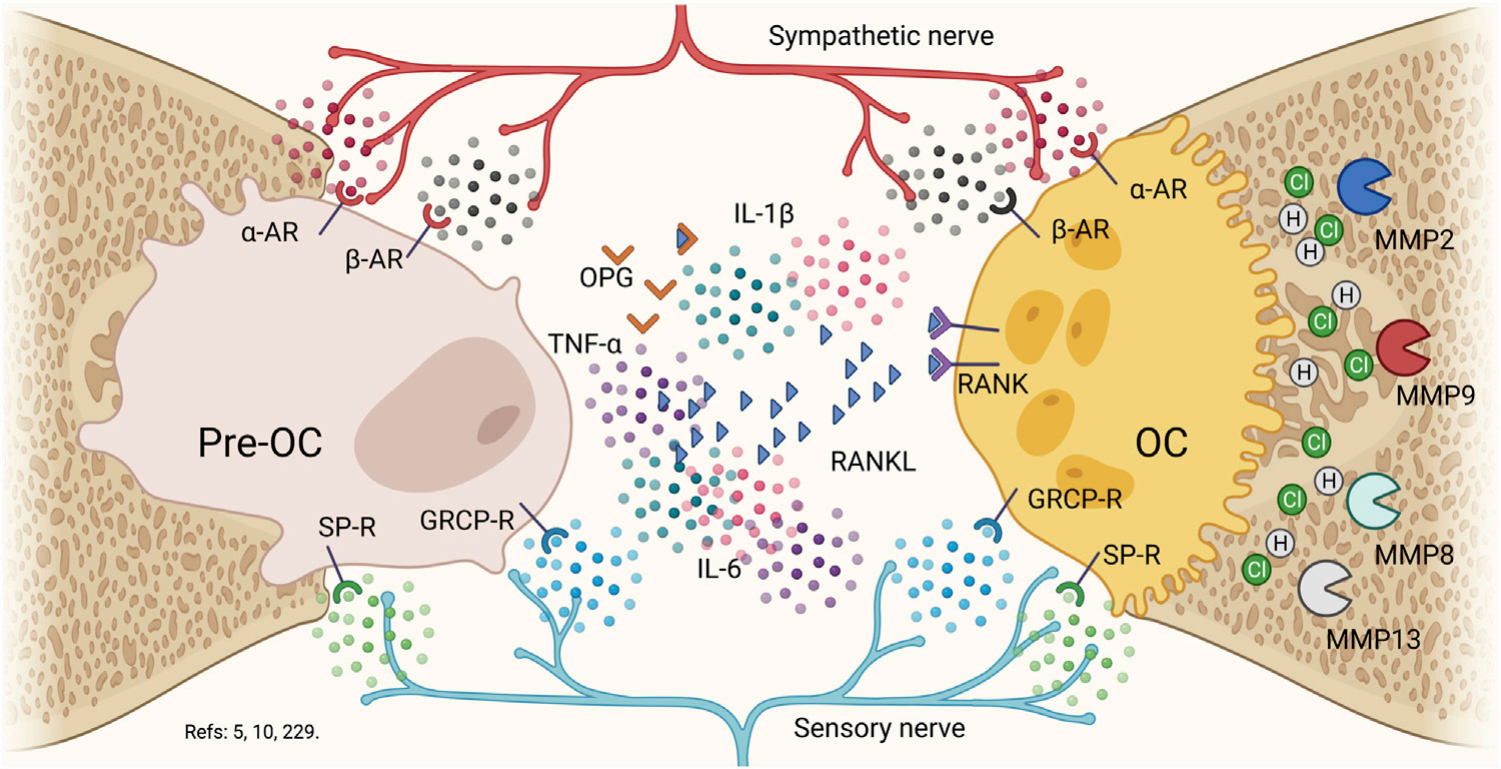
Nervous system in bone biology. Pre-osteoclast and osteoclasts possess α2-adrenergic, β2-adrenergic, substance P, and calcitonin gene related peptide surface receptors. Although, the presence of RANKL is capable of allowing the activation of osteoclasts to allow the fusion of precursors, the evidence suggests the role of epinephrine, nor-epinephrine, substance P and the calcitonin gene related peptide in regulating both of formation as bone resorption. In a pro-inflammatory context, the presence of nor-epinephrin and epinephrin will produce an increase in osteoclast function and a decrease in osteoblastic function, by decreasing the pre-osteoclast activation. On the contrary, in the presence of substance P or calcitonin gene related peptide, the effect will be higher for bone formation and bone resorption will decrease. Thus, under a distress response, the epinephrine will be activating constantly the pre-osteoclasts and osteoclasts. Osteoclasts will increase their bone-resorptive function by secreting HCl, collagenases (MMP8 and MMP13) and gelatinases (MMP2 and MMP9). Pre-OC: pre-osteoclast, PC: octeoclast, α-AR: α2-Adrenergic receptor, β-AR: β2-Adrenergic receptor: SP-R: substance P receptor, GRCP-R: gene-related with calcitonin peptide receptor, OPG. Osteoprotegerin, RANKL: Receptor of the activator of nuclear factor κB ligand, RANK: Receptor of the activator of nuclear factor κB, IL: interleukin, MMP: matrix metalloproteinases.

Interestingly, when periodontitis is induced by oral gavage with LPS in supra-physiological concentrations, the CD14 response is triggered and activates other independent TLR-TRPA pathways (Wang and Ohura, 2002). Both TRPA1 and TLR4 mediate cytokine production by NF-κB, and this factor is believed to be related to the over-expression of voltage-gated Ca^2+^ and Na^+^ channels, and possibly induce allodynia, thermal hyperplasia, or chronic pain (Huang et al., 2006). Besides, the neuron support cells were also activated by the effect of *P. gingivalis* LPS through changes in Ca^2+^ and NF-κB (Kaewpitak et al., 2020). Also, it was demonstrated that trigeminal nerves recognize the virulence factors of oral bacteria by the TLR4/CD14-MyD88-NF-κB axis (Figure 4B) (Wadachi and Hargreaves, 2005). The activation of CD14 and TLR4 stimulates phagocytosis and triggers ROS production and pro-inflammatory cytokine secretion in both immune and nervous cells (Wang and Ohura, 2002; Wadachi and Hargreaves, 2005; Diogenes et al., 2011; Díaz-Zúñiga et al., 2015b; Go et al., 2016). In this context, *P. gingivalis* has virulence factors that prevent the formation of the phage-lysosome, allowing it to survive inside the host’s cells and migrate intracellularly by both the Trojan’s horse mode or intracellular vesicles traffic (Figure 4C) (Yilmaz et al., 2004; Singh et al., 2011; Santiago-Tirado et al., 2017). Thus, the detection of *P. gingivalis* in the trigeminal ganglion, pontine nucleus of the trigeminal nerve, hippocampus, or cortex, can be due to this phenomenon. Also, the presence of human leucocytes antigen (HLA)-DR has been observed in neurilemma cells of trigeminal myelin fibers (Suzuki et al., 2015). Besides, it was reported that satellite glial cells residing in the trigeminal ganglia had an antigen-presenting cell phenotype by expressing the myeloid dendritic cell marker CD11c, the T-cell co-stimulatory molecules CD40, CD54, CD80, and CD86, and HLA-E (van Velzen et al., 2009). This finding is relevant to highlight since the infiltration of CD8^+^ lymphocytes into the trigeminal ganglion can inhibit the reactivation of herpes simplex virus type I through the release of IFN-γ and cytolytic molecules (Liu et al., 2001; Knickelbein et al., 2008). However, cytolytic molecules do not cause neuronal damage, suggesting that they are inhibited. Indeed, T cells infiltrate the trigeminal ganglion express the CD94/NKG2A complex that prevents neuronal lysis (van Velzen et al., 2009). In this way, the satellite glial cells of the trigeminal ganglion engulf microorganisms and present the antigen to both CD4^+^ and CD8^+^ T lymphocytes. However, it is unknown how these cells protect neurons from the pro-inflammatory responses induced by infiltrating T lymphocytes (van Velzen et al., 2009). In general terms, the exposed data suggest that in periodontal diseases, nerve fibers and glial cells participate in antigenic processing and presentation to secondary immune cells. The presence of dysbiosis-associated bacteria could induce a brain response, where bacteria can migrate through the trigeminal nerve, and the sympathetic response could accelerate the periodontal bone-resorptive phenomenon in the presence or the absence of pathogenic bacteria (Togari et al., 2005). In this context, there is rising evidence that a chronic sympathetic trigeminal response can accelerate the bone resorption and, at least partly, be associated with the resorptive burst under stressful conditions (Figure 5) (Togari et al., 2005). Taken together, we suggest that the existence of an Oral-Brain axis is independent of the Gut-Brain pathway and also could be in a bi-directional manner (Riviere et al., 2002; Poole et al., 2013; Dominy et al., 2019).

### Bacteria Migration Through the Lymphatic System

Regarding the lymphatic pathway, little evidence allows it to be considered a pathway for disseminating oral bacteria to the brain (Figure 4C). This hypothesis arises as an alternative when detecting oral *Treponema* in the IV brain ventricle and the cerebro-spinal fluid (CSF) (Riviere et al., 2002). Anatomically, the oral cavity and the brain would be communicated through the lymphatic system, particularly the IV ventricle and the lymph nodes of the oral cavity drain to the middle deep cervical lymph node (Louveau, 2015; Louveau et al., 2015). The teeth loss and the subsequent decrease in chewing efficiency would affect venous and lymphatic return, facilitating the entry of bacteria into the lymphatic circulation (Tsutsui et al., 2007; Nedergaard and Goldman, 2020). It is also described that aging leads to progressive dysfunction of the lymphatic vessels in the peripheral tissues. Thus, in old mice, a decrease in the diameter and coverage of the meningeal lymphatic vessels was shown compared to the young mice (Nagai et al., 2011; Da Mesquita et al., 2018). Although the migration of bacteria from the oral cavity to the brain via the lymphatics is not reported, the decrease in drainage flow could favor the chronic permanence of pathogens, but certainly, it must be exploited.

## The Oral-Gut-Brain Axis

**FIGURE 6:**
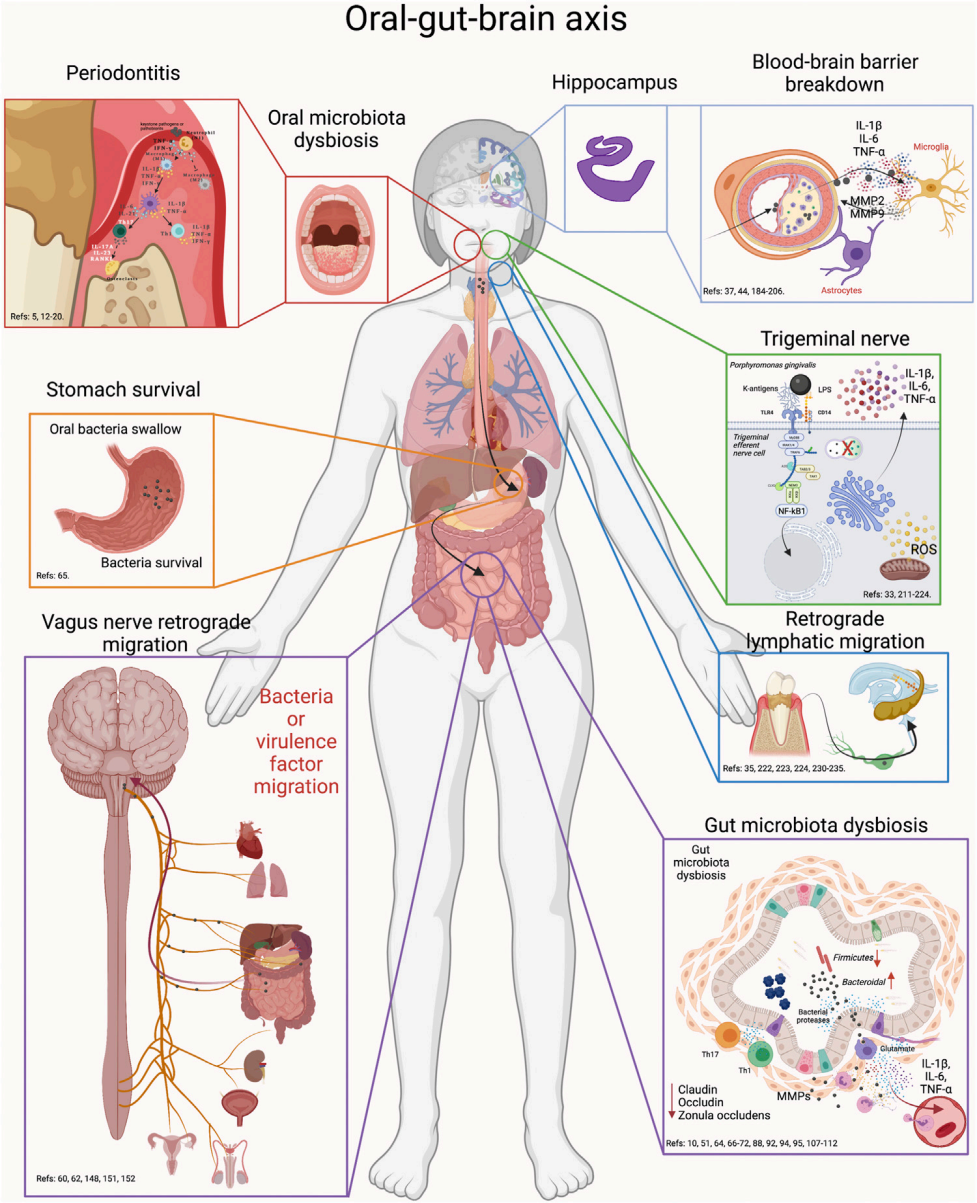
Oral-gut-brain axis. During periodontitis, the exacerbated increase in anaerobic bacteria generates alterations in the intestinal microbiota. First, swallowed oral bacteria are significantly increased. These bacteria can survive the stomach pH and upon reaching the intestine, they can cause intestinal dysbiosis, alteration in the integrity of the intestinal barrier and inflammation. This effect will cause virulence factors or pro-inflammatory mediators to diffuse into the peripheral circulation and thus migrate to the brain. In addition, oral dysbiotic bacteria can migrate to the brain via the trigeminal nerve endings or through the lymphatic vessels. Another possible route of migration is through the fibers of the vagus nerve that innervate the intestine. Thus, the possible routes of migration of oral bacteria to the brain can be through oral-brain communication or through gut-brain communication. Thus, periodontitis could be associated with neuroinflammatory events by direct communication with the brain or, indirectly, by altering intestinal homeodynamics.

No scientific study reports an association between the oral cavity, the gut, and the brain. Approaches to this possible link may lie in the existing evidence between the oral and gut microbiota with the brain through the antecedents described above (Figure 6). Also, there is rising evidence that suggests oral dysbiotic microbiota can lead a gut dysbiosis, and together, trigger neuroinflammation. When evaluating the experimental periodontitis induced by ligature, some studies detected increased levels of serum α amyloid (SAA), IL-6, vascular endothelial growth factor (VEGF), receptor of activator NF-κB ligand (RANKL), CCL5, CXCL10, and B-cell activating factor (BAFF) at the serum level (Arimatsu et al., 2014; Matsuda et al., 2015; Komazaki et al., 2017; Jia et al., 2018; Palioto et al., 2019; Hamamoto et al., 2020). Curiously, two studies demonstrated that gut microbiota dysbiosis or alteration of the intestinal epithelium’s integrity was not induced, in comparison with others that did show the presence of these phenomena (Matsuda et al., 2015; Jia et al., 2018; Palioto et al., 2019; Huang et al., 2020). An explanation for this difference may be in the observation time, while one study installed the ligature for 5 days (Palioto et al., 2019), another group did it for 10 days (Matsuda et al., 2015), and two independent research groups did it for 28 days (Jia et al., 2018; Huang et al., 2020). The evidence indicates that ligature creates measurable bone resorption and pro-inflammatory mediators from day ten onwards, together with gut microbiota dysbiosis (Garlet, 2010; Matsuda et al., 2015; Palioto et al., 2019).

Additionally, the two independent studies evaluate the association between experimental periodontitis with AD or Parkinson’s Disease-like pathologies, using different models to induce bone resorption and enough inflammatory response to trigger neurodegeneration (Feng et al., 2020; Kantarci et al., 2020; Xue et al., 2020). By characterizing gut microbiota dysbiosis induced by experimental periodontitis at day 28 post-ligation, it is possible to detect alterations in the *Firmicutes*/*Bacteroidetes* ratio, which is essential to determine intestinal inflammation (Ley et al., 2005; Koren et al., 2011; Nakajima et al., 2015). Thus, experimental periodontitis can trigger gut dysbiosis after 28 days, and the neuroinflammatory events can be related to both periodontitis and gut dysbiosis effects. However, and as we have described throughout this article, it is unclear whether periodontitis induces neuroinflammation due to oral dysbiosis, gut dysbiosis, or both.

Additionally, when evaluating the different studies that induced periodontitis through oral gavage or ligation, intending to demonstrate the presence of gut microbiota dysbiosis, it was observed that only six studies confirmed the presence of periodontitis (Arimatsu et al., 2014; Matsuda et al., 2015; Jia et al., 2018; Sato et al., 2018; Palioto et al., 2019; Huang et al., 2020). All of them demonstrated the alteration of the gut microbiota, intestinal integrity loss, or pro-inflammatory immune response. Specifically, experimental periodontitis trigger gut microbiota dysbiosis by *Clostridium*, *Firmicutes*, *Bacteroidetes* or *Lactobacillus spp* quantitative changes (Arimatsu et al., 2014; Nakajima et al., 2015; Jia et al., 2018; Sato et al., 2018; Ohtsu et al., 2019; Hamamoto et al., 2020; Huang et al., 2020; Kobayashi et al., 2020). Besides, some of them detected inflammatory mediators such as IL-1β, IL-6, IL-17, TNF-α, among others, in serum, adipose tissue, and small or long intestine, or liver (Arimatsu et al., 2014; Matsuda et al., 2015; Komazaki et al., 2017; Jia et al., 2018; Palioto et al., 2019; Hamamoto et al., 2020). When evaluating the Th17/Treg lymphocytes or M1/​​M2 macrophages ratio, only two studies analyzed the presence of these cells in the mesenteric or cervical lymph nodes and the intestine (Sato et al., 2018; Kobayashi et al., 2020). Finally, three studies evaluated the secondary effect of the application of oral bacteria, determining the presence of insulin resistance or increases in serum triglyceride levels (Arimatsu et al., 2014; Komazaki et al., 2017; Ohtsu et al., 2019). *P. gingivalis* in the intestine was associated with a higher M1/​M2 ratio, triggering receptor expressed on myeloid cells 1 (TREM1) and NF-κB expression (Kobayashi et al., 2020). The increased production of IL-1β and IL-17 produces a decrease in zo-1 and dopaminergic neurons degeneration (Ding et al., 2017; Feng et al., 2020; Kobayashi et al., 2020). Interestingly, IL-17 can cross the BBB and induce apoptotic death of dopaminergic neurons that possess the IL-17RA (Kebir et al., 2007; Arimatsu et al., 2014; Nakajima et al., 2015). IL-17A is a cytokine that allows microglia, astrocytes, and neurons to communicate constantly, and the IL-17A/IL-17RA pathway increases the production of pro-inflammatory mediators, chemokines, and antimicrobial peptides production (Chen et al., 2020). Considering that during periodontitis, the Th17 lymphocytes producing IL-17 plays a central role as the main activator of osteoclasts and in the breakdown of the BBB, the levels of IL-17 in trigeminal ganglia or hippocampus should be studied in the future (Kebir et al., 2007; Vernal et al., 2014b; Monasterio et al., 2018a; Monasterio et al., 2018b).

Given that all the models used until today use different forms and concentrations to inoculate the bacteria or induce periodontitis, it is challenging to compare them. Although all demonstrate a cognitive deterioration to a greater or lesser extent, it is debatable whether these effects are a consequence of periodontitis, bacteria, pro-inflammatory cytokines, BBB breakdown, brain bacteria colonization, or gut microbiota dysbiosis. Thus, further studies are necessary to understand the so-called Oral-Brain axis. In the present review, the collected evidence indicates that the Oral-Brain axis may be due to the bacterial-trigeminal nerve interaction through the TLR4/CD14-MyD88-NF-κB pathway, which can be driven in a bi-directional manner (Figure 4B) (Wadachi and Hargreaves, 2005; Diogenes et al., 2011; Go et al., 2016).

Also, it remains to be demonstrated if the neuroinflammation induced by experimental periodontitis is not due to gut microbiota dysbiosis. This, because both ligation and oral gavage models produce gut microbiota dysbiosis through the Oral-Gut axis (Arimatsu et al., 2014; Matsuda et al., 2015; Jia et al., 2018; Sato et al., 2018; Palioto et al., 2019; Huang et al., 2020). Although palatal inoculation would avoid the dysbiotic effect on the gut microbiota, its effects at the periodontal and brain level would be through the Oral-Brain axis (Díaz-Zúñiga et al., 2020). However, it was not demonstrated if palatal inoculation of a single bacterium induced gut microbiota dysbiosis. Future studies using experimental periodontitis models and trying to elucidate the hypothesis mentioned above should consider the measurement of bacteria or mediators at the intestinal level to confirm or rule out the gut microbiota dysbiosis.

On the other hand, it is interesting that two studies that induced periodontitis by ligation or oral gavage observed two different results (Kantarci et al., 2020; Feng et al., 2020). One of them detected microglial activation in the hippocampus and AD-like pathology, and the other detected microglial activation in the *substantia nigra pars compacta* and a PD-like pathology (Kantarci et al., 2020; Feng et al., 2020). Together, these data allow us to speculate that periodontitis may be related to proteinopathies, including AD and PD and maybe others (Kwon et al., 2020). If so, periodontitis must be reconsidered as a public health problem, being not only the leading cause of tooth loosening but also a low-grade inflammatory disease that could predispose to relevant diseases affecting the brain.

## Conclusion

The present review demonstrates that experimental periodontitis in different models can induce gut microbiota dysbiosis, affect the intestinal barrier permeability, induce an intestinal immune response, and trigger neuroinflammatory and neurodegenerative diseases. Secondly, there is evidence suggesting that oral bacteria can be recognized by trigeminal fibers and induce a response at the trigeminal ganglion level. Also, oral bacteria can trigger a sympathetic response capable of stimulating more osteoclastogenic functions.

In this way, the Oral-Gut-Brain axis could be defined based on anatomical communications, where the mouth and the intestine are in constant cross-talking. The oral-brain axis is mainly established from the trigeminal nerve and the gut-brain axis from the vagus nerve. In this context, the immune system is transversal, and an increase in inflammatory mediators at the oral or intestinal level will immediately affect brain homeostasis and vice-versa. Through the qualitative analysis of the selected papers, we observed that experimental periodontitis is capable of producing both neurodegenerative pathologies and intestinal dysbiosis, and periodontitis is likely to induce both conditions simultaneously. The severity of the neurodegenerative disease could depend, at least in part, on the effects of periodontitis in the gut microbiota, which could strengthen the immune response and create an injurious inflammatory and dysbiotic cycle. Thus, dementias would have their onset in dysbiotic phenomena that affect the oral cavity or the intestine.

### Financial Support

We thank Regional Development Program of the IADR 2021–2023 for its supply, and Fondo de Investigación de la Facultad de Odontología, FIOUCh, for the grant C019-04.
